# Supplementary material for: Life tables in entomology: A discussion on tables’ parameters and the importance of raw data
Source: PLoS One. 2024 Mar 7;19(3):e0299598. doi: 10.1371/journal.pone.0299598 (PMC10919640; doi:10.1371/journal.pone.0299598)
Supplement: S1 Fig — Crossed dot markers indicates the data points, while the solid reference line connects the first and third quartiles of the data and a dashed reference line extends the solid line to the ends of the data. (PDF) [file pone.0299598.s001.pdf]

Supplementary Figure S1

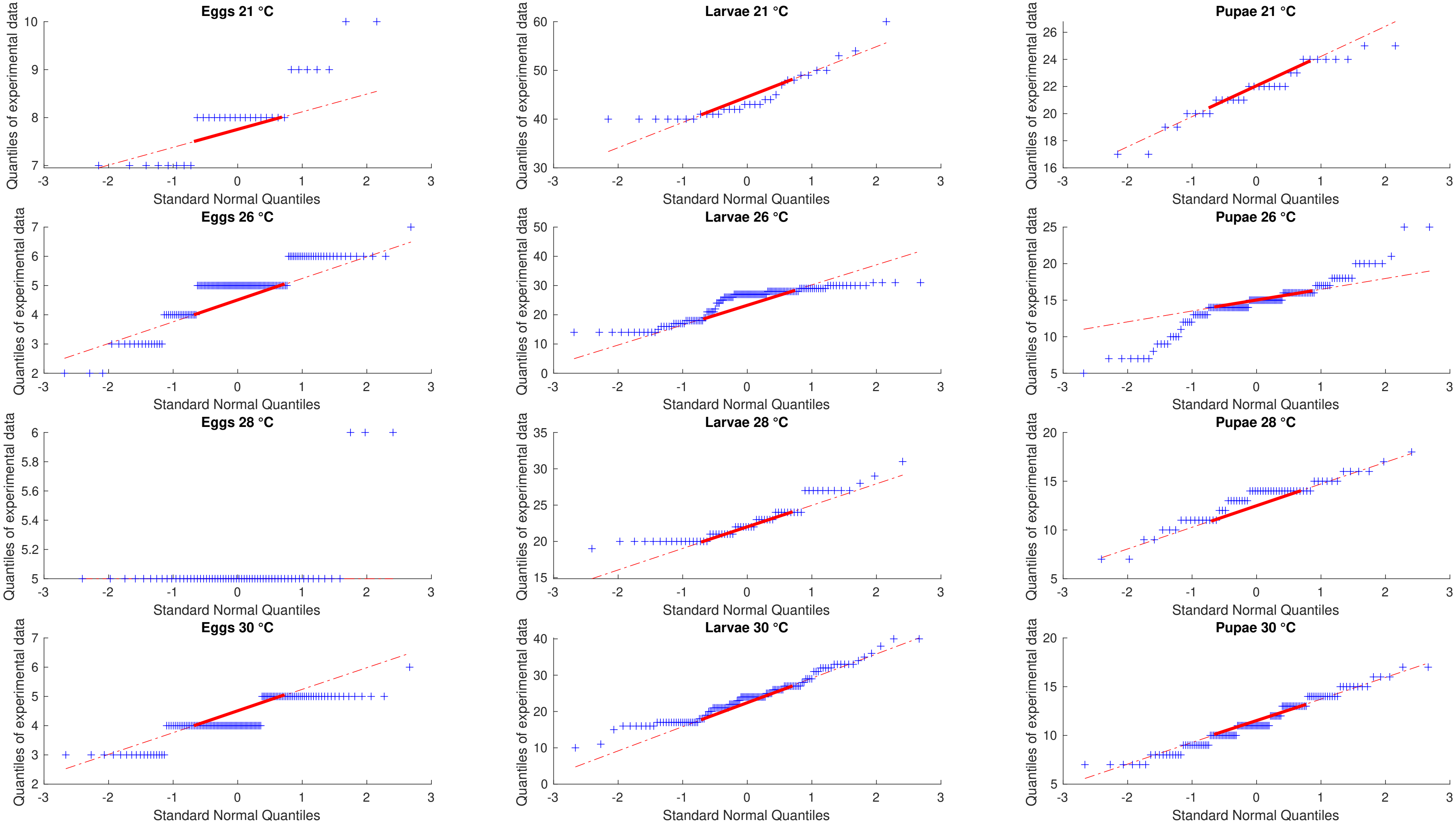

Figure S1: quantile-quantile plot of the quantiles of the quantiles of the experimental data. Crossed dot markers indicates the data points, while the solid reference line connects the first and third quartiles of the data and a dashed reference line extends the solid line to the ends of the data.
