# Supplementary material for: Life tables in entomology: A discussion on tables’ parameters and the importance of raw data
Source: PLoS One. 2024 Mar 7;19(3):e0299598. doi: 10.1371/journal.pone.0299598 (PMC10919640; doi:10.1371/journal.pone.0299598)
Supplement: Attachment — Submitted filename: Response to Reviewers.docx [file pone.0299598.s004.docx]

**Revision notes**

**Manuscript ID:** PONE-D-23-34793R1

**Title:** Life tables in entomology: a discussion on tables’ parameters and the importance of raw data.

**Journal:** PLOS ONE

**Decision letter and response to the Editor**

Dear Dr. Rossini,

Thank you for submitting your manuscript to PLOS ONE. After careful consideration, we feel that it has merit but does not fully meet PLOS ONE’s publication criteria as it currently stands. Therefore, we invite you to submit a revised version of the manuscript that addresses the points raised during the review process.

Please submit your revised manuscript by Mar 25 2024 11:59PM. If you will need more time than this to complete your revisions, please reply to this message or contact the journal office at plosone@plos.org. When you're ready to submit your revision, log on to https://www.editorialmanager.com/pone/ and select the 'Submissions Needing Revision' folder to locate your manuscript file.

Please include the following items when submitting your revised manuscript:

A rebuttal letter that responds to each point raised by the academic editor and reviewer(s). You should upload this letter as a separate file labeled 'Response to Reviewers'.

A marked-up copy of your manuscript that highlights changes made to the original version. You should upload this as a separate file labeled 'Revised Manuscript with Track Changes'.

An unmarked version of your revised paper without tracked changes. You should upload this as a separate file labeled 'Manuscript'.

If you would like to make changes to your financial disclosure, please include your updated statement in your cover letter. Guidelines for resubmitting your figure files are available below the reviewer comments at the end of this letter.

If applicable, we recommend that you deposit your laboratory protocols in protocols.io to enhance the reproducibility of your results. Protocols.io assigns your protocol its own identifier (DOI) so that it can be cited independently in the future. For instructions see: https://journals.plos.org/plosone/s/submission-guidelines#loc-laboratory-protocols. Additionally, PLOS ONE offers an option for publishing peer-reviewed Lab Protocol articles, which describe protocols hosted on protocols.io. Read more information on sharing protocols at https://plos.org/protocols?utm_medium=editorial-email&utm_source=authorletters&utm_campaign=protocols.

We look forward to receiving your revised manuscript.

Kind regards,

Ramzi Mansour

Academic Editor

PLOS ONE

Journal Requirements:

Please review your reference list to ensure that it is complete and correct. If you have cited papers that have been retracted, please include the rationale for doing so in the manuscript text, or remove these references and replace them with relevant current references. Any changes to the reference list should be mentioned in the rebuttal letter that accompanies your revised manuscript. If you need to cite a retracted article, indicate the article’s retracted status in the References list and also include a citation and full reference for the retraction notice.

[Note: HTML markup is below. Please do not edit.]

Response:

Dear Dr. Mansour,

Thank you very much for your time and for the continued interest in our manuscript PONE-D-23-34793R1 entitled “Life tables in entomology: a discussion on tables’ parameters and the importance of raw data” for publication in PLOS ONE after a minor revision.

We sincerely appreciated all the positive comments and suggestions provided by the Reviewers and a point-by-point response to all the questions is provided below this document. During the revision we carefully addressed all the suggestions, with the hope to have sufficiently increased the quality of the manuscript. We renew our availability for any further question or request, if needed. Thank you again for considering our manuscript.

Sincerely,

Luca Rossini, on behalf of the co-authors.

**Reviewers' comments**

**Reviewer 1**

**Reviewer 1:** The authors have adequately addressed my comments and (in my opinion) those of the other reviewers, and the paper can now be accepted in its revised form. I commend them on putting together a great piece of work - I'm sure this will be an important paper in the field.

**Response:** Dear Reviewer 1, thank you once again for your time dedicated to revise our manuscript. We are sincerely grateful for the very positive comment, it means much to us. We hope to reach soon the next steps in this important research, that we believe is very important to improve the data collection and sharing in Entomology, Ecology, and Biological Sciences at large.

Thank you again for helping us to improve this manuscript and for the very helpful suggestions provided during the revision.

**Reviewer 2**

**Reviewer 2:** Authors made a great effort to address all reviewers’ comment and incorporate changes in their article, which I believe are a significant improvement. I only have very minor comments below.

**Response:** Dear Reviewer 2, thank you once again for your time dedicated to revise our manuscript, and for the helpful comments and suggestions provided during the rounds of review. We sincerely appreciated the very positive comment about our study, and we hope to have reached, during this revision, a form that is acceptable for publication. A point-by-point response to all the questions follows below this document, and we hope that our changes fits with your expectations. We also renew our availability for any further change or request, if needed.

Thank you again for helping us to improve this manuscript and for the very helpful suggestions provided during the revision.

**Reviewer 2:** L25 “the benefits”

**Response:** Thank you very much for this suggestion. We have corrected the text accordingly.

**Reviewer 2:** L30 “biological aspects”.

**Response:** Thank you for this suggestion. We have corrected the text accordingly.

**Reviewer 2:** L31 avoid using “evolution” in a demographic sense. I recommend “changes in the population”, or even “demographic changes”.

**Response:** Thank you for this suggestion. We have changed the text using the suggested alternative “demographic changes”.

**Reviewer 2:** L31 “highlights this by” or “highlights this aspect by” sounded better

**Response:** Thank you for this suggestion. We have corrected the text accordingly.

**Reviewer 2:** L46 “the individuals in a population” or “from a population”

**Response:** Thank you for this suggestion. We corrected the text accordingly.

**Reviewer 2:** L49 why did you remove “a” before cohort ? Are you sure this is correct English in current form?

**Response:** Thank you for this comment. We have restored the “a” before “cohort”.

**Reviewer 2:** L54 “to obtain various information”

**Response:** Thank you for this suggestion. We have changed the text accordingly.

**Reviewer 2:** L56 “The age-stage distribution describes… that compose the insect’s life cycle.”

**Response:** Thank you for this comment. We have changed the text accordingly.

**Reviewer 2:** L76 “outside such thermal range”

**Response:** Thank you for this comment. We have modified the text accordingly.

**Reviewer 2:** L123 “this is not yet common practice, which produces a loss”

**Response:** Thank you for this suggestion. We have modified this part of the text accordingly.

**Reviewer 2:** L124 “Remarkably, the unavailability of data”. A more offline comment here: a lot of papers publish a statement similar to “data are available from authors upon request”, notably because publishing data still remains a quite tedious, non straightforward and sometimes slow process, but most importantly also costly, and the community lacks general guidelines of the multiple ways to publish data. First, while this requires lots of efforts to collect data a posteriori from authors, I wonder how often authors do respond to these post-publication queries, is it something that the authors of the present paper have a sense of ? Second, wouldn’t it also be the role of publishers and journals to build up tools and guidelines about the so many available ways to publish data beyond the gold standards (e.g., Dryad), how are all the alternative (free github repositories, as supplementary materials of a publication, etc.) valid.

**Response:** Thank you for this very interesting comment. We totally agree with you, and it is an aspect that we wanted to highlight with this paper as well. Data sharing in many fields of Life Sciences, as Entomology or Ecology, is strongly limited by the lack of a standard for the data collection and sharing. This work is a starting point for a common goal that we should achieve all together as a community. We should talk more about the problem of data sharing, and we should propose solutions in order to reach a standard, as already carried out in other fields of research. Presenting the problem is of course the first step, but much work is still needed, and we hope that the readers will support our long-term mission. Of course, Journals can enhance the data sharing, but before we need a standard. Having a common database to share entomological data would be the best option, but also if the data are published on public and free repositories (such as GitHub) following a given standard guideline would be a good option. Well, we have still much work to do and many challenges to cope, but we are sincerely glad to know that there are other researchers that have our same feelings. We found these two rounds of review very constructive not only for the feedback received on the manuscript content, but also for the personal opinions of the Reviewers, including yours, that are a source of inspiration for the future. To answer the question, we do not have a clear idea of how frequently the corresponding authors respond to requests of data sharing, but we think that it depends on the research groups. An alternative statement that we often find in many published papers is “The data are available from the authors under *reasonable* request”: well, what is “reasonable” or not is very hard to understand..!

**Reviewer 2:** L135 does it really assume “identical”, or instead “interchangeable” as random draws of a single population? A random draw does not assume identity, but representativeness. The distribution will be imperfect (unrealistic) in case the draw is too small relative to the size of the population, and in case the population itself is not representative of the species. I’m not very sure I agree with the following argument L135-137; bootstrapping will smooth-out the distribution and a distribution is always based on individuals’ traits, this last sentence as currently written seems to oppose bootstrapping to individual-based data, so I don’t understand…

**Response:** Thank you very much for this comment. We have modified the lines 135-137 accordingly, to make this part of the text clearer. The bootstrap technique hides the actual distribution of the development times, this was the meaning behind this part of the text. We hope that now the sentence is clearer and that our change fits with your expectations.

**Reviewer 2:** L147 “of insect population models”, I assume not all modellers deal with pests.

**Response:** Thank you for this suggestion. We have changed the text accordingly.

**Reviewer 2:** L148 “exclusive usage of life tables’ synthetic information”

**Response:** Thank you for this suggestion. We have modified the text accordingly.

**Reviewer 2:** L200 is it instead “implies that individuals from a single population can have two different developmental times” ?

**Response:** Thank you for this suggestion. Yes, the meaning is correct, and the sentence was modified accordingly.

**Reviewer 2:** L316 “are both publicly”

**Response:** Thank you for this suggestion. We have modified this sentence accordingly.

**Reviewer 2:** L369 “Furthermore, any synthetic parameters from descriptive statistics will always come with some loss of information”.

**Response:** Thank you for this suggestion. We have modified this sentence accordingly.
